# 

**Published:** 1879-02

**Authors:** 


### CONTENTS.

ORIGINAL,

ART. I.—On the treatment of Chronic Catarrh of the Bladder, and some forms of Acute Cystitis. By Theodore Deecke, M. D., Special Pathologist, New York State Lunatic Asylum, Utica, N. Y. .... 241

ART. II.—Abstract of the Proceedings of the Buffalo Medical Association. By Joseph Fowler, M. D., Secretary .... 247

ART. III.—Medical Society of the County of Albany. By T. K. Perry, M. D., Secretary. 259

MISCELLANEOUS,

The Therapeutic Value of Iodoform, .... 266
Poisonous Nature of Ozone, .... 272
On Diphtheria, .... 273
Damiana (*Turnera Aphrodisiaca*), .... 273
Application of the Shellac Cloth or Poroplastic Spinal Jacket, .... 274

EDITORIAL.

Medicine and Hygiene, .... 274
Death in Buffalo of Dr. Heman P. Babcock, of Oakland, California, .... 276
New Journals .... 277
Telephonic Communication, .... 277
Maltine and its Combinations, .... 278
Lactopeptine, .... 278

BOOKS REVIEWED:

Reports of the Surgeon-General, .... 278
Cell Doctrine. By James Tyson, M. D., .... 278
Medical and Surgical Uses of Electricity. By George M. Beard, A. M., M. D., and A. D. Rockwell, A. M., M. D., .... 279
Diseases of the Bladder and Urethra in Women. By Alexander J. C. Skene, M. D., .... 279
Contributions to Reparative Surgery. By Gurdon Buck, M. D., .... 279

Books and Pamphlets Received, .... 280

